# Comparison of clinical results between high grade patterns in stage I lung adenocarcinoma

**DOI:** 10.1111/1759-7714.14578

**Published:** 2022-07-12

**Authors:** Hyun Woo Jeon, Young‐Du Kim, Sung Bo Sim, Mi Hyoung Moon

**Affiliations:** ^1^ Department of Thoracic and Cardiovascular Surgery, Bucheon St. Mary's Hospital, College of Medicine The Catholic University of Korea Seoul Republic of Korea; ^2^ Department of Thoracic and Cardiovascular Surgery, Seoul St. Mary's Hospital, College of Medicine The Catholic University of Korea Seoul Republic of Korea

**Keywords:** adenocarcinoma, lung cancer, micropapillary, solid

## Abstract

**Background:**

The histological subtype has been introduced in invasive lung adenocarcinoma. The predominant micropapillary and solid subtypes are categorized as high‐grade patterns and provide a worse prognosis. However, the prognostic analysis of high‐grade patterns has not previously been fully investigated. Thus, this study aimed to investigate the prognostic role of high‐grade patterns in pathological stage I lung adenocarcinoma.

**Methods:**

Patients with stage I lung adenocarcinoma and micropapillary or solid components were reviewed. Clinicopathological features and clinical course were compared in these subtypes, and prognostic factors were analyzed in high‐grade patterns.

**Results:**

The patients were classified into five groups based on the presence of micropapillary or solid subtypes, namely, micropapillary predominant, solid predominant, both nonpredominant subtypes, only minor micropapillary subtype, and only minor solid subtype present. Disease‐free interval was significantly different, and the micropapillary predominant group showed worse disease‐free interval (*p* = 0.001). Contrastingly, the solid predominant group showed significantly worse overall survival among high‐grade patterns (*p* = 0.035). The multivariate analysis revealed an association between smoking, micropapillary predominant, blood vessel invasion, and visceral pleural invasion with recurrence and more association between solid predominant and visceral pleural invasion with overall survival.

**Conclusions:**

Clinical results were different in stage I high‐grade adenocarcinoma. The predominant micropapillary subtype is the independent prognostic factor for recurrence. However, the solid subtype is the significant factor for overall survival. Furthermore, the predominant subtype is the most valuable and independent prognostic factor for predicting recurrence or survival.

## Graphical abstract

Micropapillary and solid subtype were considered as high grade patterns because of poor prognosis. However, clinical results are different between high grade groups. Micropapillary showed more recurrence and solid subtype is more associated with overall survival.

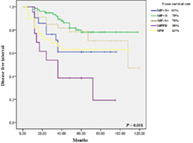

## INTRODUCTION

Adenocarcinoma is most common in lung cancer.^1^ The most prominent feature is pathological heterogeneous.^2^ A new classification of lung adenocarcinoma, according to the histological subtype, has been adopted by the World Health Organization (WHO) in 2015.^3^ Lung adenocarcinoma has been categorized by the predominant pattern after histological subtype quantification in 5% increments because adenocarcinoma varies in terms of histological subtype and proportion. The prognosis is significantly different according to the histological subtype despite the same stage. Lepidic predominant subtype as low‐grade has a favorable result and acinar and papillary subtypes are intermediate grade. Micropapillary (MP) or solid predominant (SP) subtypes as high‐grade patterns have worse prognosis, despite curative resection in early‐stage lung adenocarcinoma.^4^ High‐grade patterns are associated with smoking, lymphatic vessel invasion (LVI), and nonground‐glass opacity (GGO) lesions, which are prognostic factors in lung cancer.^5^ Furthermore, high‐grade patterns showed early lymph node (LN) metastasis, higher metabolic activity, visceral pleural invasion, and spread through air spaces (STAS).^6^, ^7^, ^8^ Additionally, the presence of these high‐grade patterns without a predominant subtype also shows unfavorable results.^9^, ^10^ However, these studies were analyzed in comparison with non‐MP or solid subtypes and some studies demonstrated that MP or solid nonpredominant components did not show a worse prognosis.^11^ A few studies were found on prognostic analysis in lung adenocarcinoma among MP and solid subtypes. We hypothesized that MP and solid subtypes have different clinicopathological features despite categorized high‐grade patterns. The present study reviewed patients with MP or solid subtypes, including predominant and nonpredominant subtypes after curative resection in stage I lung adenocarcinoma. Additionally, the clinicopathological features were investigated and the prognostic factors were analyzed.

## METHODS

**FIGURE 1:**
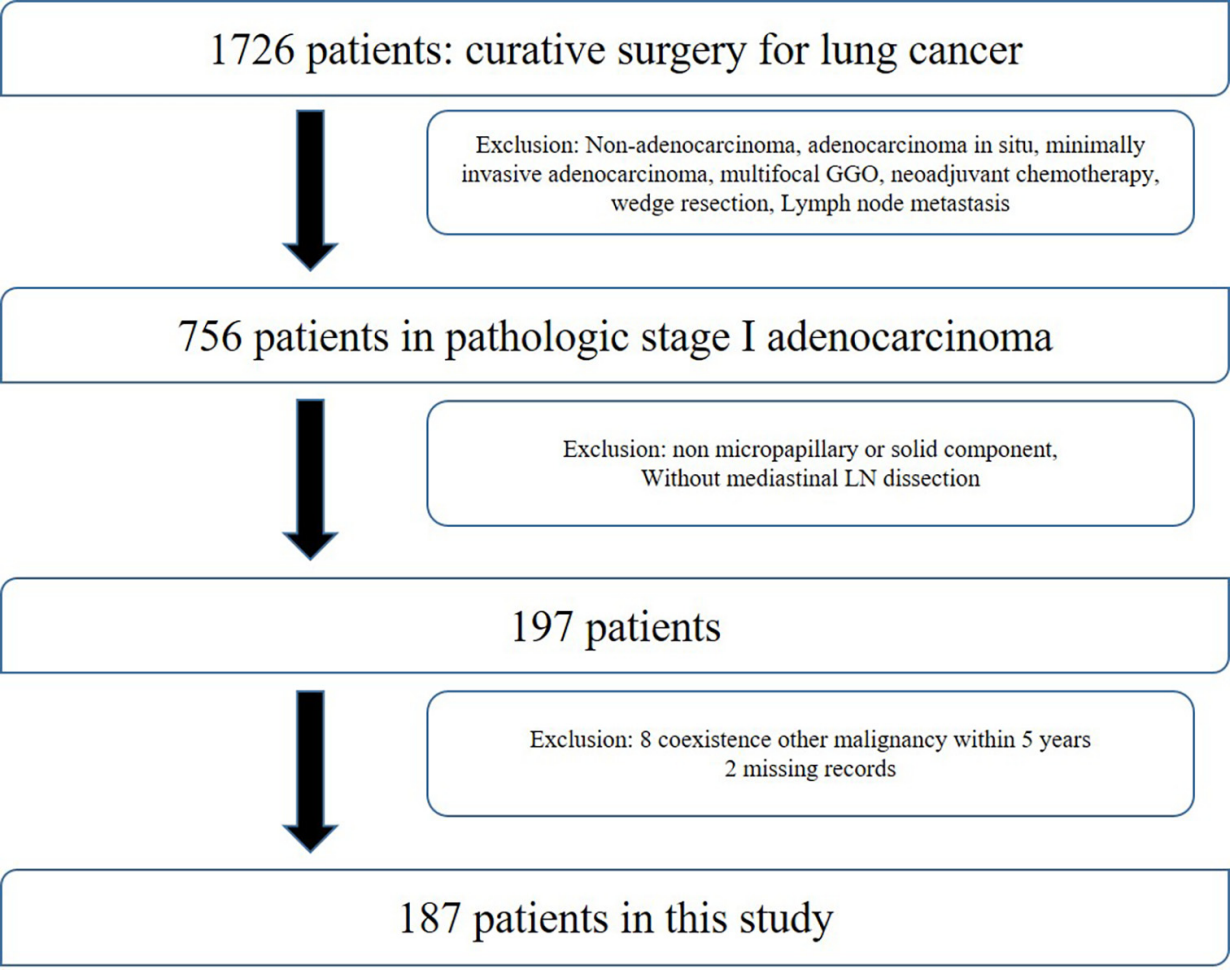
Diagram of patient selection

We reviewed the electronic medical records of patients who underwent curative resection for invasive lung adenocarcinoma from January 2010 to April 2017 at Seoul and Bucheon St. Mary's Hospital. This retrospective study was conducted with the approval of the Institutional Review Board of the Catholic Medical Center (Republic of Korea). Written informed consent from the patients was waived because of the retrospective nature of the study. We classified the pathological stage of patients according to the eighth edition of the tumor‐node‐metastasis (TNM) classification. Patients with pathological stage I were included in the study. Among these patients, those with adenocarcinoma in situ, minimally invasive adenocarcinoma, multifocal GGO, neoadjuvant chemotherapy, and missing medical records were excluded, as well as patients with incomplete resection, wedge resection, and perioperative death. Additionally, patients without mediastinal node evaluation during surgery and patients with coexistence of other malignancies were also excluded except for a curative state with 5‐year disease‐free survival. Patients with histological subtypes were classified based on the pathological report. Patients with MP or solid predominant subtype were first included. Patients with the presence of MP or solid components were included according to the histological subtype. Finally, a total of 187 patients with predominant or minor subtypes of high‐grade patterns were reviewed (Figure 1).

Preoperative assessments included blood sampling, including carcinoembryonic antigen (CEA), pulmonary function test, chest computed tomography (CT), echocardiography, positron emission tomography‐CT (PET‐CT), brain magnetic resonance imaging, bone scanning, and bronchoscopy.

We reviewed the CEA level and feature of the main tumor on chest CT, which was a solid mass or part‐solid nodule. The maximum standardized uptake value (SUVmax) was reviewed on PET‐CT.

Patients were classified into the five groups by the proportion of MP or solid subtype as follows: (1) MP predominant (MPP), (2) solid predominant (SP), (3) MP and solid components but not predominant (MP+/S+), (4) only MP components but not predominant (MP+/S−), (5) only solid components but not predominant (MP−/S +).

Follow‐up (F/U) was conducted every 3 months for 1 year after the operation, every 4 months in the second year, and every 6 months thereafter. Chest CT evaluation was conducted on every visit. All patients were followed until recurrence and death or loss of F/U. Recurrence was defined as local or extrathoracic metastasis based on clinical and pathological evidence. Overall survival (OS) was defined as the interval from operation to the date of death, or final follow‐up visit. Disease‐free interval (DFI) was defined as the interval from operation to the date of local or distant metastasis.

### Statistical analysis

All statistical analyses were conducted using the statistical package for the social sciences version 18 (SPSS Inc.). Clinicopathological factors were compared according to the high grade patterns. Continuous variables were compared using the Kruskal–Wallis test, and categorical variables were compared using the chi‐square test.

The survival curves of DFI and OS were conducted using the Kaplan–Meier method and log‐rank test was used to identify the differences among these groups.

Multivariate analysis was used to assess the effect of the covariates on DFI and OS using the Cox proportional hazards model after checking the proportionality assumption. Variables with *p‐*values of <0.05 in the univariate analysis were included in the multivariate analysis.

## RESULTS

Patient characteristics are demonstrated in Table 1. The median age of patients was 64 years (range, 36–85). Patients included 92 (49.2%) males, and patients who were current smokers or with a smoking history accounted for 69 (36.9%). The preoperative level of CEA was median 2 (range, 0.49–25.83). Preoperative CT identified part‐solid nodules in 60 (32.1%) patients. No pure GGOs were identified. PET‐CT revealed a median SUVmax of 5 (range, 0.8–17.6).

**TABLE 1:** Baseline patient characteristics

Characteristic	Total (*n* = 187)
	Median (range) or n (%)
Age	64 (36–85)
Male	92 (49.2)
Smoking	69 (36.9)
CEA	2 (0.49–25.83)
PET SUVmax	5 (0.8–17.6)
Mixed GGO	60 (32.1)
Lobectomy	172 (92)
Segmentectomy	15 (8)
VATS	173 (92.5)
Thoracotomy	14 (7.5)
Number of dissected LN	12 (3–47)

Data are presented as the median (minimum‐maximum) or frequencies and percentages as appropriate.

Abbreviations: CEA, carcinoembryonic antigen; GGO, ground‐glass opacity; LN, lymph node; SUVmax, maximum standardized uptake value; VATS, video‐assisted thoracic surgery.

The pathological data (Table 2) revealed a median tumor size of 2.3 cm (range, 0.7–4). Well differentiated tumors were rare. Acinar predominant tumors were the most common histological subtype (50.8%). Meanwhile, the lepidic predominant subtype was rare despite its early‐stage (8.6%). MPP and SP were determined in 13 and 42 patients, respectively (7% and 22.5%). Recently, a new grading system has been proposed for lung adenocarcinoma, and a cutoff value of 20% in patients with high‐grade patterns had worse prognoses.^12^ In this grading system for investigation, MP components of ≥ 20% were identified in 50 (26.7%) patients and solid patterns of ≥ 20% in 67 (35.8%) patients. LVI was identified in 95 (50.8%) patients, blood vessel invasion (BVI) in 32 (17.1%), and visceral pleural invasion (VPI) in 47 (25.1%). A total of 122 patients were pathologically staged as IA (65.2%) and 65 (34.8%) as stage IB. Platinum‐based adjuvant chemotherapy was conducted in 13 (6.9%) patients.

**TABLE 2:** Pathological data

Characteristic	Total (*n* = 187)
	Median (range) or n (%)
Size	2.3 (0.7–4)
Differentiation	
Well	20 (10.7)
Moderate	110 (58.8)
Poor	57 (30.5)
Predominant subtype	
Acinar	95 (50.8)
Papillary	20 (10.7)
Lepidic	16 (8.6)
MP	13 (7)
Solid	42 (22.5)
MP ≥ 20%	50 (26.7)
Solid ≥ 20%	67 (35.8)
Distance from the resected margin	3 (0.5–9.20)
LVI	95 (50.8)
BVI	32 (17.1)
VPI	47 (25.1)
pStage IA	122 (65.2)
pStage IB	65 (34.8)
Adjuvant treatment	13 (6.9)

Data are presented as the median (minimum‐maximum) or frequencies and percentages as appropriate.

Abbreviations: BVI, blood vessel invasion, LVI, lymphatic vessel invasion, MP, micropapillary; VPI, visceral pleural invasion.

Patients were classified into the five groups by the proportion of MP or solid subtype (Table 3). Age was not significantly different in the five groups (*p* = 0.633). The SP group revealed more male predominance (*p* = 0.001) compared with other groups, and smoking (*p* = 0.055) and poor differentiation (*p* < 0.001) were also more associated with SP. The MP+/S− group had the lowest median value of SUVmax (*p* < 0.001), which was more associated with mixed GGO lesion (*p* < 0.001) and well differentiation (*p* < 0.001). The lepidic predominant subtype was more common in the MP+/S− group (*p* = 0.078).

**TABLE 3:** The groups according to the proportion of micropapillary and solid subtype

	MP+/S+ (*n* = 21)	MP+/S− (*n* = 77)	MP−/S+ (*n* = 34)	MPP (*n* = 13)	SP (*n* = 42)	*p*‐value
Age	64 (38–80)	64(38–82)	61 (36–85)	63 (51–80)	67 (42–82)	0.633
Male	12 (57)	30 (39)	12 (32.4)	6 (46.2)	32 (76.2)	0.001
Smoking	8 (38.1)	20 (26)	15 (40.5)	4 (30.8)	22 (52.4)	0.055
CEA	1.76 (0.5–5.59)	1.71 (0.49–19.15)	2.69 (0.5–25.83)	2.28 (0.85–6)	1.97 (0.5–19.13)	0.013
SUVmax	5.47 (2.1–14.55)	3.5 (0.8–17.6)	5.8 (1.4–14.6)	6.5 (2.1–17.4)	6.82 (1.7–17.14)	< 0.001
Mixed GGO	5 (23.8)	38 (49.4)	10 (27)	2 (15.4)	5 (11.9)	< 0.001
Lobectomy	18 (85.7)	69 (89.6)	32 (94.1)	13 (100)	40 (95.2)	0.455
Size	2.5 (1.5–3.9)	2 (0.8–4)	2.45 (0.9–4)	3.3 (0.7–4)	2.25 (0.9–4)	0.011
Differentiation						
Well	0 (0)	19 (24.7)	0 (0)	0 (0)	1 (2.4)	< 0.001
Poorly	8 (38.1)	9 (11.7)	7 (20.6)	4 (30.8)	29 (69.1)	< 0.001
Predominant subtype						
Acinar	15 (71.4)	51 (66.2)	29 (85.3)	0 (0)	0 (0)	< 0.001
Lepidic	2 (9.5)	11 (14.3)	3 (8.8)	0 (0)	0 (0)	0.078
LVI	13 (61.9)	38 (49.4)	20 (58.8)	9 (69.2)	15 (35.7)	0.109
BVI	4 (19)	9 (11.7)	8 (23.5)	2 (15.4)	9 (21.4)	0.522
VPI	6 (28.6)	17 (22.1)	8 (23.5)	3 (23.1)	13 (31)	0.854
EGFR	9 (42.9)	48 (62.3)	18 (52.9)	6 (46.2)	11 (26.2)	0.005
Stage IB	10 (47.6)	15 (19.5)	15 (44.1)	9 (61.5)	17 (40.5)	0.004
Adjuvant Tx	1 (4.8)	2 (2.6)	1 (2.9)	3 (23.1)	6 (14.3)	0.018

Data are presented as the median (minimum‐maximum) or frequencies and percentages as appropriate.

Abbreviations: BVI, blood vessel invasion; CEA, carcinoembryonic antigen; GGO, ground‐glass opacity; LVI, lymphatic vessel invasion; SUVmax, maximum standardized uptake value; VPI, visceral pleural invasion.

The MPP group showed the largest tumor size at that time of operation (*p* = 0.011). However, LVI, BVI, and VPI showed no significant difference among the five groups. For the epidermal growth factor receptor (EGFR), the SP group was less associated with EGFR expression compared with other groups (*p* = 0.005) (Table 4).

**TABLE 4:** Univariate and multivariate analysis for recurrence

Variables	Univariate			Multivariate		
	HR	95% CI	*p‐*value	HR	95% CI	*p‐*value
Smoking	2.172	1.279–3.690	0.004	2.440	1.423–4.184	0.001
SUVmax	1.088	1.023–1.156	0.007			
Non‐GGO (solid mass)	2.696	1.355–5.366	0.005			
MPP	3.413	1.665–6.996	0.001	4.136	1.982–8.631	<0.001
MP ≥ 20%	2.338	1.366–4.002	0.002			
BVI	2.571	1.431–4.618	0.002	2.533	1.393–4.607	0.002
VPI	2.208	1.279–3.813	0.004	2.083	1.187–3.658	0.011

Abbreviations: BVI, blood vessel invasion; GGO, ground‐glass opacity; MP, micropapillary; MPP, micropapillary predominant; SUVmax, maximum standardized uptake value; VPI, visceral pleural invasion.

**FIGURE 2:**
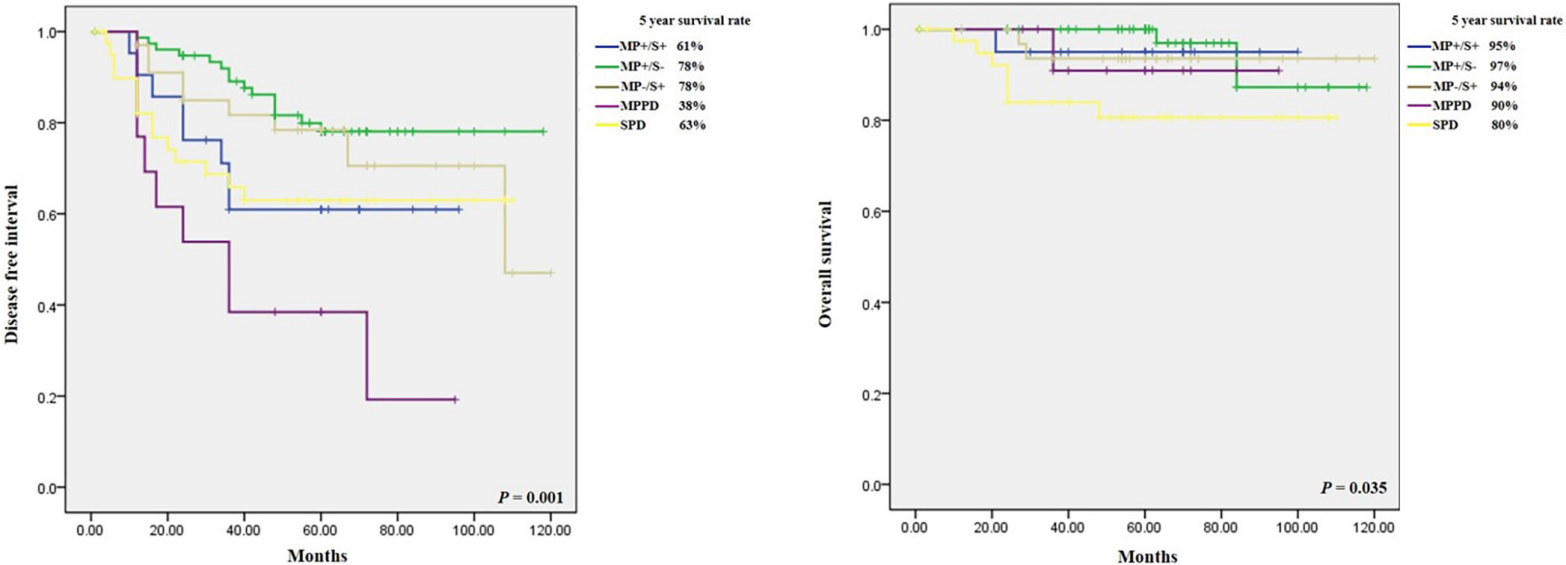
Survival curve for disease free interval and overall survival

During the F/U period (median F/U of 60 months), 55 (29.4%) recurrences and 13 (7%) deaths were identified, according to the survival curve for DSI and OS (Figure 2). The cause of death was cancer‐related. A significant difference was found in DFI, and recurrence was the most common in the MPP group (*p* = 0.001). However, the SP group was the most associated with death for OS (*p* = 0.035) (Table 5).

**TABLE 5:** Univariate and multivariate analysis for survival

Variables	Univariate			Multivariate		
	HR	95% CI	*p‐*value	HR	95% CI	*p‐*value
Age	1.076	1.008–1.149	0.028			
Smoking	4.974	1.526–16.215	0.008	3.752	1.117–12.608	0.032
SUVmax	1.128	1.002–1.270	0.046			
SP	4.786	1.606–14.264	0.005	3.308	1.071–10.219	0.038
VPI	4.910	1.603–15.039	0.005	4.013	1.292–12.468	0.016

Abbreviations: SUVmax, maximum standardized uptake value; SP, solid predominant; VPI, visceral pleural invasion.

Of the 55 patients with recurrence, bone metastasis was identified in seven patients. Pleural metastasis was identified in 10 patients. Lung to lung metastasis was noted in 15 patients. LN metastasis was in eight patients. Two patients had recurrence from the bronchial stump with mediastinal LN metastasis. The other patients showed distant metastasis. A total of 49 patients received chemotherapy, radiotherapy, or tyrosine kinase inhibitor (TKI). Others refused treatment. The MP+/S+ group showed that eight patients had a postoperative recurrence. EGFR expression was determined in four patients, one patient received TKI, and seven patients had chemo‐ or radiotherapy. A total of 15 patients showed recurrence in the MP+/S− group. Among them, 12 showed EGFR expression and 10 received TKI. A total of nine patients showed recurrence in the MP−/S+ group. EGFR expression was identified in four patients. Chemoradiotherapy was conducted in four patients and TKI treatment in three patients. A total of nine patients showed recurrence in the MPP. EGFR expression was identified in four patients and three patients were treated with TKI and patients with chemoradiotherapy. A total of 14 patients showed recurrence in SP. EGFR expression was determined in seven patients, six patients were given TKI and seven underwent chemoradiotherapy.

The univariate analysis for DFI revealed that smoking (*p* = 0.004), SUVmax (*p* = 0.007), non‐GGO lesion (*p* = 0.005), MPP (*p* = 0.001), MP of ≥ 20% (*p* = 0.002), BVI (*p* = 0.002), and VPI (*p* = 0.012) were associated with DFI. Multivariate analysis revealed that smoking (*p* = 0.001), MPP (*p* < 0.001), BVI (*p* = 0.002), and VPI (*p* = 0.011) were significant prognostic factors for DFI (Table 4).

The survival analysis for OS revealed that age (*p* = 0.028), smoking (*p* = 0.008), SUVmax (*p* = 0.046), SP (*p* = 0.005), and VPI (*p* = 0.005) were associated with OS using the univariate analysis. The multivariate analysis revealed that smoking (*p* = 0.032), SP (*p* = 0.038), and VPI (*p* = 0.016) were significant prognostic factors for OS (Table 5).

## DISCUSSION

Lung adenocarcinoma is the most common in non‐small cell lung cancer, and early‐stage lung adenocarcinoma is the best option for surgical resection, with a curative goal.^13^ However, lung cancer is one of the worst prognostic diseases and is the leading cause of death.^14^ Stage IA lung adenocarcinoma has the most survival postoperative benefit. However, clinical results remain unsatisfactory, and the 5‐year survival rate is approximately 70%–90%. The prognostic factor analysis is important to predict recurrence and conduct effective treatment. The TNM staging system is a well‐known classification for lung cancer. However, predicting the prognosis in early‐stage lung adenocarcinoma has many limitations. A new histological subtype classification was proposed by the International Association for the study of lung cancer/American thoracic society/European Respiratory Society in 2011.^15^ The WHO adopted this proposal for lung adenocarcinoma in 2015.^3^ According to this classification, invasive lung adenocarcinoma is classified into five histological subtypes (lepidic, acinar, papillary, MP, and solid), and this classification was based on the predominant histological subtype after histological subtype quantification in 5% increments because lung adenocarcinoma has heterogeneous histological subtypes and a wide variety of histological subtype distribution and proportion.

Since the introduction of this proposal, numerous studies have been conducted to predict the prognosis according to the predominant histological subtype, nonpredominant histological subtype, or proportion of each histological subtype.

The lepidic predominant pattern shows tumor cells along with alveolar wall preservation. The lepidic predominant pattern of <5 mm stromal or vascular invasion has previously been categorized as minimally invasive adenocarcinoma with excellent survival. Lepidic predominant invasive lung adenocarcinoma has also been reported to have favorable outcomes as a low‐grade malignancy. Invasive lung adenocarcinoma has mixed subtypes, and a favorable prognosis may be associated with the lepidic subtype proportion.^16^, ^17^

Solid or MP predominant subtypes are known as high‐grade malignancies in invasive lung adenocarcinoma.^18^ Many studies have been found on solid or MP predominant subtypes, which are associated with poor prognosis. Glandular differentiation was absent in the SP pattern, without acinar, papillary, or lepidic patterns. Ujiie et al. investigated the prognosis of the SP subtype and found that the SP subtype is an independent prognostic factor for early recurrence after curative resection in stage I lung adenocarcinoma.^19^ One meta‐analysis revealed that the SP subtype is associated with worse outcomes with lung adenocarcinoma even if curative resection was conducted.^20^

The MP predominant subtype has been added to the new histological classification since 2011. The MP pattern consists of small papillary clusters of glandular cells in the air space and does not show fibrovascular cores in the pathological view. Cell adhesion is decreased so MP subtype induced more metastasis and a worse prognosis. The MP predominant subtype is associated with spread through air spaces (STAS), which has been found to be a significant prognostic factor in lung adenocarcinoma.^21^

Furthermore, numerous studies have indicated that a minimal proportion of high‐grade patterns have worse prognoses. Zhao et al. investigated the prognostic impact of minor components of MP and solid subtypes^22^ and indicated that minor components of high‐grade patterns were independent prognostic factors for LN metastasis, recurrence‐free survival, and OS. Chang et al. investigated the metabolic activity of minor components of high‐grade patterns and revealed a higher SUVmax in minor components of high‐grade patterns, which were associated with LN metastasis. LN metastasis became more common despite the small‐sized lung adenocarcinoma with high‐grade patterns.^23^

In a summary of previous studies, MP or solid histological subtypes have worse prognoses although these subtypes are not predominant.^24^ However, these statistical results came from the analysis in comparison with nonhigh‐grade patterns. We wondered whether the clinical course is the same between two high‐grade patterns despite of different clinicopathologfical. In our study, 55 patients with high‐grade patterns experienced recurrence after curative resection in stage I lung adenocarcinoma. The recurrence rate was relatively high (29.4%). Yoshizawa et al. conducted a validation study for adenocarcinoma classification with East Asian patients^25^ and found that the 5‐year DFS of MP predominant (0%) was significantly lower than the 5‐year DFS of patients with solid subtype (43.3%). This result is exactly analogous to our study result. In our study, the 5‐year DFI rate of the MP predominant subtype was only 38%, which is the independent prognostic factor for recurrence, and the tumor size of the MP predominant subtype was larger than other subtypes at that time of surgery. Yoshizawa et al. reported that MP and SP subtype were not distinct for OS, although DFS for MP was much lower than the SP subtype. *EGFR* mutations were more frequent in the MP predominant subtype than in the solid subtype and survival was increased because of more EGFR‐TK susceptibility. Warth et al. indicated that post recurrence survival was significantly decreased in the SP subtype,^26^ which was rarely associated with *EGFR* mutations. This means that the effects of TKIs are unsatisfactory. However, in the recurrence in our study, the frequency of *EGFR* mutations and TKI treatment was similar between the MP predominant and the SP subtypes. The worse result was that TKI response is much lower in the SP subtype despite *EGFR* expression mutations.^27^

Some studies have conducted survival analysis according to the proportion of high‐grade patterns and demonstrated worse clinical results according to the increased proportion of high‐grade patterns.^28^ However, the proportion of each subtype did not reach the significance for DFI and OS in our study. In our study, we conclude that the predominant subtype is the most powerful risk factor in the survival analysis. Furthermore, only the nonpredominant MP components showed better clinical outcomes than other subtypes and were associated with more GGO features, well‐differentiated, lepidic predominant, and *EGFR* mutations.

This study had certain limitations, including its retrospective nature and small sample size. MP or solid subtypes are rare in stage I lung adenocarcinoma. However, we focused on the comparison of clinicopathological features and clinical outcomes between two high‐grade patterns, which was the aim of this study. Comparing previous studies, overall survival was relatively high. Only 13 patients died. The reason for this may be the patients that were lost to follow‐up in the study. Nowadays, segmentectomy is a surgical option for lesions less than 2 cm. In our study, incidence of segmentectomy was low. Segmentectomy is one of the surgical options in GGO dominant lesions. However, there were no GGO dominant lesions in this study. Finally, this study was not conducted from multiple centers; thus, selection bias may be inevitable.

In conclusion, MP and SP subtypes showed different clinical courses despite being high‐grade patterns after curative resection in pathological stage I lung adenocarcinoma. The MP predominant subtype increased in recurrence and the SP subtype decreased in survival. Therefore, a larger study will be needed to determine the prognostic factors among the high‐grade patterns.

## CONFLICT OF INTEREST

All authors declare that they have no conflicts of interest associated with this study.

## References

[1] Ferlay J , Steliarova‐Foucher E , Lortet‐Tieulent J , Rosso S , Coebergh JWW , Comber H , et al. Cancer incidence and mortality patterns in Europe: estimates for 40 countries in 2012. Eur J Cancer. 2013;49:1374–403. 23485231 10.1016/j.ejca.2012.12.027

[2] Tsao MS , Marguet S , Le Teuff G , et al. Subtype classification of lung adenocarcinoma predicts benefit from adjuvant chemotherapy in patients undergoing complete resection. J Clin Oncol. 2015;33:3439–46. 25918286 10.1200/JCO.2014.58.8335 PMC4606061

[3] Travis WD , Brambilla E , Burke AP , Marx A , Nicholson AG . WHO Classification of Tumours of the Lung, Pleura, Thymus and Heart. 4th ed. Lyon: International Agency for Research on Cancer; 2015. 10.1097/JTO.0000000000000663 26291007

[4] Yanagawa N , Shiono S , Abiko M , et al. The clinical impact of solid and micropapillary patterns in resected lung adenocarcinoma. J Thorac Oncol. 2016;11:1976–83. 27374456 10.1016/j.jtho.2016.06.014

[5] Mäkinen JM , Laitakari K , Johnson S , Mäkitaro R , Bloigu R , Pääkkö P , et al. Histological features of malignancy correlate with growth patterns and patient outcome in lung adenocarcinoma. Histopathology. 2017;71:425–36. 28401582 10.1111/his.13236

[6] Yu Y , Jian H , Shen L , Zhu L , Lu S . Lymph node involvement influenced by lung adenocarcinoma subtypes in tumor size ≤3 cm disease: a study of 2268 cases. Eur J Surg Oncol. 2016;42:1714–9. 27017272 10.1016/j.ejso.2016.02.247

[7] Nakamura H , Saji H , Shinmyo T , Tagaya R , Kurimoto N , Koizumi H , et al. Close association of IASLC/ATS/ERS lung adenocarcinoma subtypes with glucose‐uptake in positron emission tomography. Lung Cancer. 2015;87:28–33. 25481488 10.1016/j.lungcan.2014.11.010

[8] Terada Y , Takahashi T , Morita S , KAshiwabara K , Nagayama K , Nitadori JI , et al. Spread through air space is an independent predictor of recurrence in stage III (N2) lung adenocarcinoma. Interact Cardiovasc Thorac Surg. 2019;29(3):442–8. 31106332 10.1093/icvts/ivz116

[9] Chen T , Luo J , Gu H , Gu Y , Huang Q , Wang Y , et al. Impact of solid minor histologic subtype in postsurgical prognosis of stage I lung adenocarcinoma. Ann Thorac Surg. 2018;105:302–8. 29162222 10.1016/j.athoracsur.2017.08.018

[10] Zhao Y , Wang R , Shen X , Pan Y , Cheng C , Li Y , et al. Minor components of micropapillary and solid subtypes in lung adenocarcinoma area predictors of lymph node metastasis and poor prognosis. Ann Surg Oncol. 2016;23:2099–105. 26842488 10.1245/s10434-015-5043-9 PMC4858562

[11] Roh MS , Lee JI , Choi PJ , Hong YS . Relationship between micropapillary component and micrometastasis in the regional lymph nodes of patients with stage I lung adenocarcinoma. Histopathology. 2004;45:580–6. 15569048 10.1111/j.1365-2559.2004.01953.x

[12] Moreira AL , Ocampo PSS , Xia Y , Zhong H , Russell PA , Minami Y , et al. A grading system for invasive pulmonary adenocarcinoma: a proposal from the international association for the study of lung cancer pathology committee. J Thorac Oncol. 2020;15:1599–610. 32562873 10.1016/j.jtho.2020.06.001 PMC8362286

[13] Goya T , Asamura H , Yoshimura H , Kato H , Shimokata K , Tsuchiya R , et al. Prognosis of 6644 resected non‐small cell lung cancers in Japan: a Japanese lung cancer registry study. Lung Cancer. 2005;50:227–34. 16061304 10.1016/j.lungcan.2005.05.021

[14] Siegel RL , Miller KD , Jemal A . Cancer statistics, 2019. CA Cancer J Clin. 2019;69:7–34. 30620402 10.3322/caac.21551

[15] Travis WD , Brambilla E , Noguchi M , Nicholson AG , Geisinger KR , Yatabe Y , et al. International association for the study of lung cancer/american thoracic society/european respiratory society international multidisciplinary classification of lung adenocarcinoma. J Thorac Oncol. 2011;6:244–85. 21252716 10.1097/JTO.0b013e318206a221 PMC4513953

[16] Makinen JM , Laitakari K , Johnson S , Makitaro R , Bloigu R , Lappi‐Blanco E , et al. Nonpredominant lepidic pattern correlates with better outcome in invasive lung adenocarcinoma. Lung Cancer. 2015;90:568–74. 26506915 10.1016/j.lungcan.2015.10.014

[17] Jeon HW , Kim YD , Sim SB , Moon MH . Prognostic impact according to the proportion of the lepidic subtype in stage IA acinar‐predominant lung adenocarcinoma. Thorac Cancer. 2021;12:2072–7. 34033216 10.1111/1759-7714.14013 PMC8287017

[18] Zheng M . Classification and pathology of lung cancer. Surg Oncol Clin. 2016;25(3):447–68. 10.1016/j.soc.2016.02.003 27261908

[19] Ujiie H , Kadota K , Chaft JE , Buitrago D , Sima CS , Lee MC , et al. Solid predominant histologic subtype in resected stage I lung adenocarcinoma is an independent predictor of early, extrathoracic, multisite recurrence and of poor postrecurrence survival. J Clin Oncol. 2015;33(26):2877–84. 26261257 10.1200/JCO.2015.60.9818 PMC4554749

[20] Miyahara N , Nii K , Benazzo A , Hoda MA , Iwasaki A , Klepetko W , et al. Solid predominant subtype in lung adenocarcinoma is related to poor prognosis after surgical resection: a systematic review and meta‐analysis. Eur J Surg Oncol. 2019;45(7):1156–62. 30772108 10.1016/j.ejso.2019.01.220

[21] Lee JS , Kim EK , Kim M , Shim HS . Genetic and clinicopathologic characteristics of lung adenocarcinoma with tumor spread through air spaces. Lung Cancer. 2018;123:121–6. 30089582 10.1016/j.lungcan.2018.07.020

[22] Zhao Y , Wang R , Shen X , Pan Y , Cheng C , Li Y , et al. Minor Components of Micropapillary and Solid Subtypes in Lung Adenocarcinoma are Predictors of Lymph Node Metastasis and Poor Prognosis. Ann Surg Oncol. 2016;23:2099–105. 26842488 10.1245/s10434-015-5043-9 PMC4858562

[23] Chang C , Sun X , Zhao W , Wang R , Qian X , Lei B , et al. Minor components of micropapillary and solid subtypes in lung invasive adenocarcinoma (3 cm): PET/CT findings and correlations with lymph node metastasis. Radiol Med. 2020;125:257–64. 31823295 10.1007/s11547-019-01112-x

[24] Morales‐Oyarvide V , Mino‐Kenudson M . High‐grade lung adenocarcinomas with micropapillary and/or solid patterns: a review. Curr Opin Pulm Med. 2014;20:317–23. 24852329 10.1097/MCP.0000000000000070

[25] Yoshizawa A , Sumiyoshi S , Sonobe M , Kobayashi M , Fujimoto M , Kawakami F , et al. Validation of the IASLC/ATS/ERS lung adenocarcinoma classification for prognosis and association with EGFR and KRAS gene mutations: analysis of 440 Japanese patients. J Thorac Oncol. 2013;8(1):52–61. 10.1097/JTO.0b013e3182769aa8 23242438

[26] Warth A , Muley T , Meister M , Stenzinger A , Thomas M , Schirmacher P , et al. The novel histologic International Association for the Study of Lung Cancer/American Thoracic Society/European Respiratory Society classification system of lung adenocarcinoma is a stage‐independent predictor of survival. J Clin Oncol. 2012;30:1438–46. 22393100 10.1200/JCO.2011.37.2185

[27] Yoshida T , Ishii G , Goto K , Yoh K , Niho S , Umemura S , et al. Solid predominant histology predicts EGFR tyrosine kinase inhibitor response in patients with EGFR mutation‐positive lung adenocarcinoma. J Cancer Res Clin Oncol. 2013;139(10):1691–700. 23974272 10.1007/s00432-013-1495-0 PMC11824609

[28] Zombori T , Nyári T , Tiszlavicz L , Pálföldi R , Csada E , Géczi T , et al. The more the micropapillary pattern in stage I lung adenocarcinoma, the worse the prognosis‐a retrospective study on digitalized slides. Virchows Arch. 2018;472(6):949–58. 10.1007/s00428-018-2337-x 29611055

